# Heat inactivation of clinical COVID-19 samples on an industrial scale for low risk and efficient high-throughput qRT-PCR diagnostic testing

**DOI:** 10.1038/s41598-022-06888-z

**Published:** 2022-02-21

**Authors:** Oona Delpuech, Julie A. Douthwaite, Thomas Hill, Dhevahi Niranjan, Nancy T. Malintan, Hannah Duvoisin, Jane Elliott, Ian Goodfellow, Myra Hosmillo, Alexandra L. Orton, Molly A. Taylor, Christopher Brankin, Haidee Pitt, Douglas Ross-Thriepland, Magdalena Siek, Anna Cuthbert, Ian Richards, John R. Ferdinand, Colin Barker, Robert Shaw, Cristina Ariani, Ian Waddell, Steve Rees, Clive Green, Roger Clark, Abhishek Upadhyay, Rob Howes

**Affiliations:** 1 Discovery Biology, Discovery Sciences, R&D, AstraZeneca, Cambridge, UK; 2 Bioscience, Research and Early Development, Oncology R&D, AstraZeneca, Cambridge, UK; 3 In Vivo Expressed Biologics, Discovery Sciences, R&D, AstraZeneca, Cambridge, UK; 4 Biologics Engineering, Research and Early Development, Oncology R&D, AstraZeneca, Cambridge, UK; 5 Charles River Laboratories, Chesterford Research Park, Saffron Walden, CB10 1XL UK; 6 Division of Virology, Department of Pathology, Addenbrooke’s Hospital, Cambridge, UK; 7 Animal Science and Technologies, BioPharmaceuticals R&D, AstraZeneca, Cambridge, UK; 8 Facilities Management, BioPharmaceuticals R&D, AstraZeneca, Cambridge, UK; 9 Clinical Operations, Late-Stage Development, Oncology R&D, AstraZeneca, Cambridge, UK; 10 Department of Medicine, University of Cambridge, Cambridge, UK; 11 BiologIC Technologies Ltd., Soham, UK; 12 Oral Product Development, Pharmaceutical Technology and Development, Operations, AstraZeneca, Macclesfield, UK; 13 Wellcome Sanger Institute, Hinxton, UK; 14 BioPharmaceuticals R&D, AstraZeneca, Cambridge, UK

**Keywords:** High-throughput screening, Infectious-disease diagnostics

## Abstract

We report the development of a large scale process for heat inactivation of clinical COVID-19 samples prior to laboratory processing for detection of SARS-CoV-2 by RT-qPCR. With more than 266 million confirmed cases, over 5.26 million deaths already recorded at the time of writing, COVID-19 continues to spread in many parts of the world. Consequently, mass testing for SARS-CoV-2 will remain at the forefront of the COVID-19 response and prevention for the near future. Due to biosafety considerations the standard testing process requires a significant amount of manual handling of patient samples within calibrated microbiological safety cabinets. This makes the process expensive, effects operator ergonomics and restricts testing to higher containment level laboratories. We have successfully modified the process by using industrial catering ovens for bulk heat inactivation of oropharyngeal/nasopharyngeal swab samples within their secondary containment packaging before processing in the lab to enable all subsequent activities to be performed in the open laboratory. As part of a validation process, we tested greater than 1200 clinical COVID-19 samples and showed less than 1 Cq loss in RT-qPCR test sensitivity. We also demonstrate the bulk heat inactivation protocol inactivates a murine surrogate of human SARS-CoV-2. Using bulk heat inactivation, the assay is no longer reliant on containment level 2 facilities and practices, which reduces cost, improves operator safety and ergonomics and makes the process scalable. In addition, heating as the sole method of virus inactivation is ideally suited to streamlined and more rapid workflows such as ‘direct to PCR’ assays that do not involve RNA extraction or chemical neutralisation methods.

## Introduction

Coronavirus disease (COVID-19) is caused by a novel coronavirus, SARS-CoV-2, that emerged in Wuhan City, China in December 2019. SARS CoV-2 is transmitted via the inhalation of aerosolized virus and via unprotected contact through fomites. The median incubation period for the virus is usually 5 days but may range from 1 to 14 days^1–3^. In the early stages of the pandemic, while most infected people have a mild illness, some individuals require hospitalisation and intensive care treatment^4,5^. The UK detected its first cases of COVID-19 in late February 2020, with transmission attributed to person-person contact by international travellers^5^. Thereafter, the disease rapidly spread through international travel corridors and locally through community transmissions^6^. The rapid spread of SARS CoV-2 and significant harm to human health led to the UK government introducing infection control and prevention measures; including mass testing of the population, isolation of infected individuals and restrictions on people movement in general.

In April 2020, the Cambridge COVID-19 Testing Centre (CCTC) was set up to perform qPCR-based detection of SARS CoV-2 in oropharyngeal/nasopharyngeal (OP/NP) swab samples, to support the UK’s national testing capacity^7^. The approved CCTC process detects the SARS-Cov-2 ORF1ab gene by RT-qPCR in RNA extracted from clinical OP/NP swab samples. In this standard protocol, sample tubes carrying potentially viable SARS-Cov-2 were removed from their secondary containment, checked and transferred into sample racks. Following this an aliquot of viral transport media (VTM) is manually transferred to a deep-well assay plate, and mixed with an RNA extraction kit lysis buffer. Viral inactivation occurs at the point of lysis buffer addition, as a result of its guanidine isothiocyanate content, followed by an additional heating step of 65 °C for 10 min in a small dry air laboratory oven for inactivation of any viral material on the sample plate outer surfaces. These steps are manually performed within a microbiological safety cabinet (MSC) in a Containment Level 2 laboratory. These requirements makes the process expensive and resource intensive, due to the cost of MSCs, their running and maintenance costs, and PPE requirements. The process is also time consuming due to the requirement for strict techniques for handling high hazard samples. Working in a MSC for the extended periods of time required in a high throughput testing laboratory also leads to operator discomfort and a decline in performance.

Heat has been widely used for the inactivation of various viruses^8^ by denaturing the secondary structure of the capsid, resulting in impaired functions of the virus through reduced ability to attach to and thus replicate in a host cell^9,10^. Several studies have demonstrated that SARS-CoV-2 and other coronaviruses such as SARS and MERS, can be effectively inactivated using heat^8,11–15^. Such studies have demonstrated that 65 °C for 10 min or 95 °C for 3 min resulted in viral inactivation, however the use of 95 °C for 3 min also resulted in decreased sensitivity in the downstream RT-qPCR assay (2.2 Cq increase)^8^. Others have also observed that at some temperatures, the sensitivity of the RT-qPCR assay is affected, interfering with the diagnosis of patient samples^8,16^ while in a different study, treatment of 70 °C for up to 30 min did not appear to affect Cq values in nasopharyngeal samples^13^. Therefore a balance between viral inactivation and reduced detection by RT-qPCR must be achieved for any heating protocol.

Here we describe a significant change to the standard workflow by the development of a heat inactivation (HI) protocol for clinical OP/NP swab samples within their original UN3373 secondary containment that enables the HI of 500 swab samples/hour per oven and all subsequent laboratory processing on the open bench.

## Results

### Heat inactivation of the murine hepatitis virus (MHV), a surrogate for human SARS-CoV-2

Given the hazard classification of the SARS-CoV-2 as a Hazard Group 3 pathogen by the Advisory Committee on Dangerous Pathogens (ACDP), a surrogate model mouse hepatitis coronavirus (MHV-A59)^17^, was chosen for viral inactivation studies. MHV is a prototype of the group II coronavirus (beta-coronavirus) in common with severe acute respiratory syndrome coronaviruses such as SARS-CoV-2^18^. Commonly found in laboratory mice, MHV-A59 is a Hazard Group 1 pathogen, can be replicated to high titers in cell culture and has been used as an experimental model to understand the biology of coronaviruses^17–21^. Furthermore, MHV-A59 is a widely accepted coronavirus surrogate model and has been used to assess the survival of coronaviruses in the environment as well as sensitivity to biocidal agents and heat^21^.

To examine the effect of heat on virus viability, we prepared high titer cell-free MHV-A59 samples in the same VTM as used in clinical samples. Samples were subjected to heat inactivation in a thermal cycler for 5, 10 or 15 min at 60 °C, 65 °C and 75 °C, followed by a tissue culture infectious dose (TCID_50_) assay to measure sample viability. In the absence of heat treatment, high levels of infectious virus were observed in the TCID_50_ assay for samples containing cell-free virus of 4.88 × 10^4^ TCID_50_/ml (average of 3 experiments; 6.3 × 10e^4^, 6.3 × 10e^4^ and 2.0 × 10e^4^). Following heat treatment, no live virus was detected under any of the conditions examined while the control conditions confirmed virus infectivity**.** This demonstrates that heat alone was sufficient to render high titers of the surrogate viral samples non-infectious.

### Large-scale inactivation of SARS-CoV-2 in OP/NP clinical samples from COVID-19 patients and the general public

**Figure 1:**
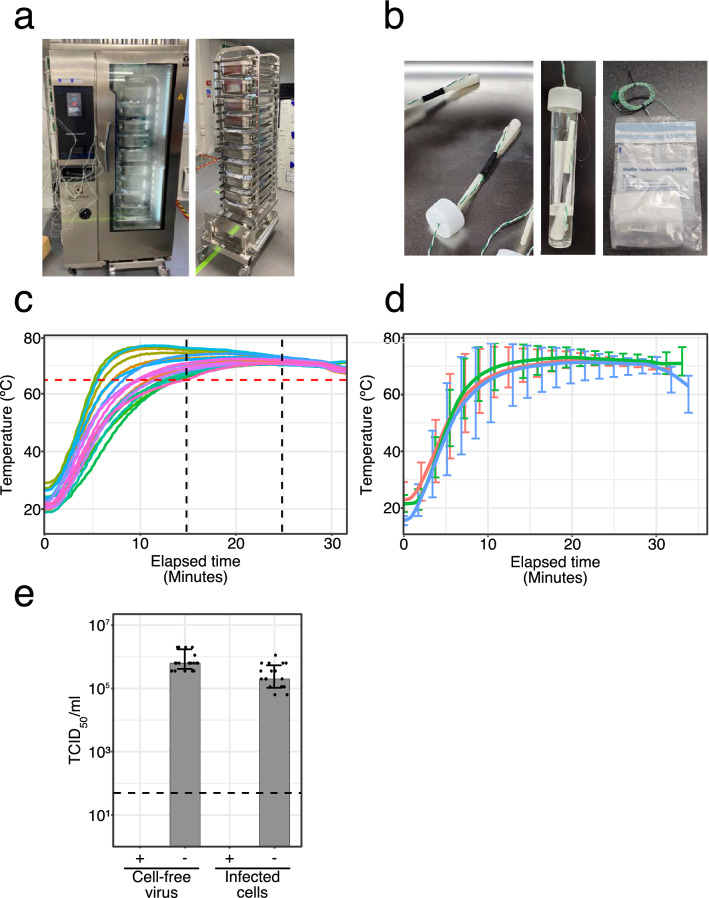
Validation of the industrial steam oven for bulk HI. (**a**) The Electrolux Skyline Combi Oven and the trolley loading system. The oven is shown in temperature validation mode with thermocouple wires in place. Under normal operation these are not included. (**b**) Thermocouple swab vials used for measuring temperature at the point of the swab to determine exposure of potential viable virus to heat. (**c**) An example heat mapping study showing the thermal profile of 20 thermocouple swab vials placed throughout the oven, 2 in each of 10 trays, and including 250 samples in total to mimic a full oven. Each coloured line represents an individual thermocouple swab vial (**d**) Average of three heat mapping data sets of 20 thermocouple, including that shown in (**c**). Vertical bars represent the temperature range shown for every 10th data point. (**e**) Inactivation of the SARS-CoV-2 surrogate virus MHV-A59 in the Electrolux Skyline Combi Oven using the temperature profile shown in (**c**) and (**d**), showing reduction in infectivity in human cells. Data is plotted as mean + /− standard deviation. The dashed line represents the limit of detection of the assay. Data was plotted using R (v4.1.0 “Camp Pontanezen”) with ggplot2 (v3.3.3) and the figure was assembled using Affinity Designer (1.9.3).

A wet heating system from the catering industry (Electrolux Skyline Combi Oven) was identified for the bulk HI of 500 samples per hour while held within their UN3373 secondary containment. Efficient heat penetration to all samples throughout the oven was achieved by the use of a racking system comprising 25 sample bags per tray, with 10 trays per oven, loaded on a roll-in/roll-out trolley system for easy handling (Fig. 1a and Supplementary Fig. S1). Experiments were performed to optimise a heating cycle to achieve a sustained temperature of at least 65 °C for 10 min for all of the samples with minimal overheating in a time efficient manner. Twenty temperature probes were packed inside mock OP/NP sample vials along with a swab, and packed in a manner mimicking clinical samples (Fig. 1b). These thermocouple swab vials along with and an additional 230 unused OP/NP swab test kits, assembled as if used, were placed in the oven, with the thermocouple swab vials distributed 2 per tray throughout the oven. The most optimal heating cycle in terms of achieving a minimum heat exposure of 65 °C or above for at least 10 min in all thermocouple swab vials was identified and confirmed in three independent experiments. Using the optimised parameters (Supplementary Table 1), it took approximately 15 min for all samples to reach 65 °C, and no sample exceeded 80 °C (Fig. 1, d and Supplementary Fig. S2). The overall cycle time was 30 min, meaning 500 samples could be subject to the bulk HI protocol per hour per oven.

Next, a range of different OP/NP vial and swab combinations were tested using the thermocouple swabs created to reflect the diversity of test kits used across the COVID-19 testing network (Supplementary Fig. S3). This showed that all of the OP/NP vial and swab types tested achieved the same required heat inactivation conditions, and did not exceed 80 °C (Supplementary Fig. S3). Additionally, as the CCTC also analysed pooled OP/NP tests supplied by the University of Cambridge containing up to 10 swabs in a single tube^22^, we also confirmed that vials containing multiple OP/NP swabs performed similarly to vials containing single swabs. We observed a 4 min time difference before reaching the required 65 °C, but no additional over-heating (Supplementary Fig. S4).

Having established a suitable temperature profile for HI of OP/NP swab vials in bulk using the industrial steam oven, we then confirmed viral inactivation in this precise setting using the surrogate virus MHV-A59. Clinical samples typically contain a mix a cell free virus and virus infected epithelial cells. We therefore prepared mock OP/NP swab viral samples containing either cell-free MHV-A59 viral particles or MHV-A59 infected 17Cl-1 cells in VTM. Swab vials were packaged in UN3373 secondary containment before treatment according to the bulk HI protocol in the industrial steam oven. As before, TCID_50_ assays confirmed the absence of infectious virus in all of the MHV-A59 samples following heat treatment. Two independent experiments with multiple biological repeats were performed and identical results were obtained (Fig. 1e). Thus, the bulk HI protocol comprising of a 30 min cycle achieving a 10 min hold at 65 °C or greater, reduces virus infectivity by up to 6 log10 TCID_50_/ml.

### Detection of SARS-CoV-2 in clinical OP/NP samples following heat exposure

As shown in previous experiments, efficient bulk HI of samples for a minimum of 10 min heat treatment at 65 °C required a temperature ramping programme whereby samples typically reached temperatures of 75–80 °C for around 20 min. The nature of the bulk HI process means some sample to sample variability in heat exposure is unavoidable. We therefore examined the effect of heat and duration of the heat step on assay sensitivity in clinical OP/NP swab samples. Randomly selected clinical OP/NP swab samples received at the CCTC were retained following standard testing^7^ and reserved at 4 °C until their RT-qPCR data was available. SARS-CoV-2 positive samples were then identified and used for experiments to examine a range of temperatures and incubation times using a thermal cycler or a small dry air oven. Samples were re-tested in the RT-qPCR assay and Cq values compared to control conditions of heat inactivation at 65 °C for 10 min, reflecting the HI step of the standard CCTC assay protocol. The control conditions were included instead of comparison to the clinical test result to avoid time-induced changes.

An initial study was performed to understand the intrinsic variability in our assay when retesting known SARS-CoV-2 positive samples, by repeating replicate tests of a panel of samples on three consecutive days (Supplementary Fig. S5). Using an analysis of variance to estimate the pooled within-sample variability we calculated a variance of 0.33 Cq. This value was used as a reference point to determine whether treatment-based differences of subsequent experiments were within or outside the expected assay variability.

**Figure 2:**
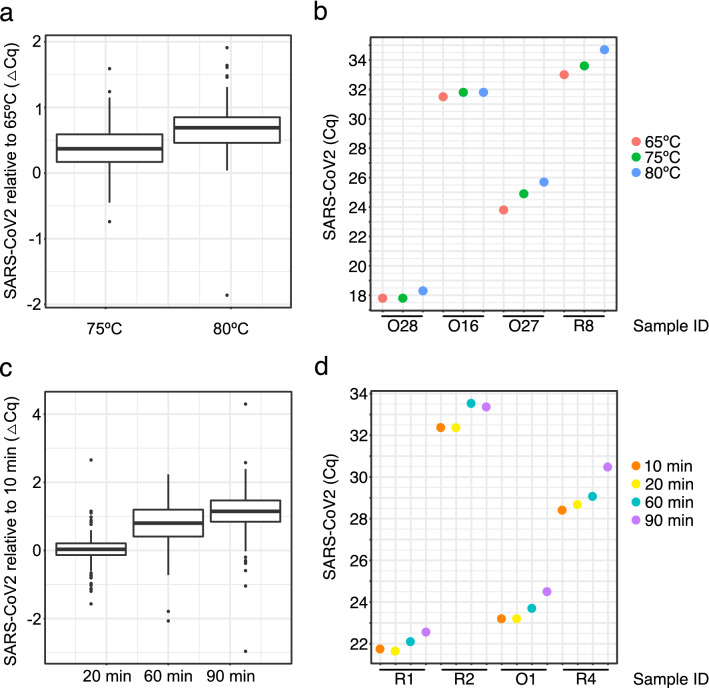
SARS-CoV-2 detection by RT-qPCR following heat treatment. (**a**) Impact on SARS-CoV-2 RT-qPCR Cq value in OP/NP samples following incubation at 70 °C and 80 °C compared to 65 °C for 10 min. (**b**) Selected samples from (**a**) showing the effect is not Cq dependent. (**c**) Impact on SARS-CoV-2 RT-qPCR Cq value in OP/NP samples following incubation at 65 °C for 20, 60 and 90 min compared to 10 min. (**d**) Selected samples from (**c**) showing the effect is not Cq dependent. See Supplementary Tables 2 and 3 for summary statistics. Data was plotted using R (v4.1.0 “Camp Pontanezen”) with ggplot2 (v3.3.3) and the figure was assembled using Affinity Designer (1.9.3).

We then compared incubation temperatures of 75 °C and 80 °C for 10 min using SARS-CoV-2 positive clinical samples chosen in the Cq range of 17 to 33. Incubation at 75 °C for 10 min had little effect on SARS-CoV-2 detection, with a 0.39+/− 0.4 Cq variation which is in line with the known assay variability of 0.33 Cq (Fig. 2a and Supplementary Table 2). Incubation of the same samples at 80 °C for 10 min however resulted in a small loss in sensitivity (i.e. a higher Cq) for SARS-CoV-2 detection by RT-qPCR with a mean increase in Cq value of 0.69+/− 0.55 (Fig. 2a and Supplementary Table ). Within the OP/NP swab samples tested at different temperatures, some were more affected than others. This variation was independent of the level of SARS-CoV-2 detection (i.e. the Cq value). For example in Fig. 2b, sample O28 and O16 (initial Cq values of 18 and 32 respectively) show no effect of heat, while samples O27 and R8 (initial Cq values of 24 and 33 respectively) show a 2 Cq difference in SARS Cov-2 detection.

Next, we evaluated the effect of incubation time at a constant temperature of 65 °C using, SARS-CoV-2 positive clinical samples chosen in the Cq range of 21 to 36 Cq. Incubation of samples for 20 min showed no effect on SARS-CoV-2 detection, seen as a mean change in Cq value of (0.01+/− 0.5), however 60 and 90 min incubation showed an increase in Cq value of up to 2 Cq (Fig. 2c and Supplementary Fig. S6). This increase in Cq is above the intra assay variability of 0.33Cq and therefore indicates some loss of sensitivity for these longer incubation times (Supplementary Table 3). Similar to the observations on temperature range, the impact appears to be sample-dependent as opposed to Cq-dependent. For example, samples R1 and R2 having Cq values of 22 and 34 respectively, show only a 1Cq variation with time, while samples O1 and R4 having Cq values of 23 and 28 respectively, show over a 2 Cq variation with time (Fig. 2d and Supplementary Table 2).

In summary, higher temperatures and longer incubation times compared to the existing HI protocol of 10 min at 65 °C result in higher Cq values in the RT-qPCR assay. The degree to which the Cq value is shifted appears to be sample-specific rather than Cq-specific. The most likely explanation being variation in OP/NP sample quality such as the presence of RNAses which may be activated during HI, leading to degradation of SARS-CoV-2 RNA^1^. Importantly, the detection of SARS-CoV-2 in all positive samples is maintained.

### Validation of a bulk HI protocol for use in an approved clinical SARS-CoV-2 diagnostic assay

**Figure 3:**
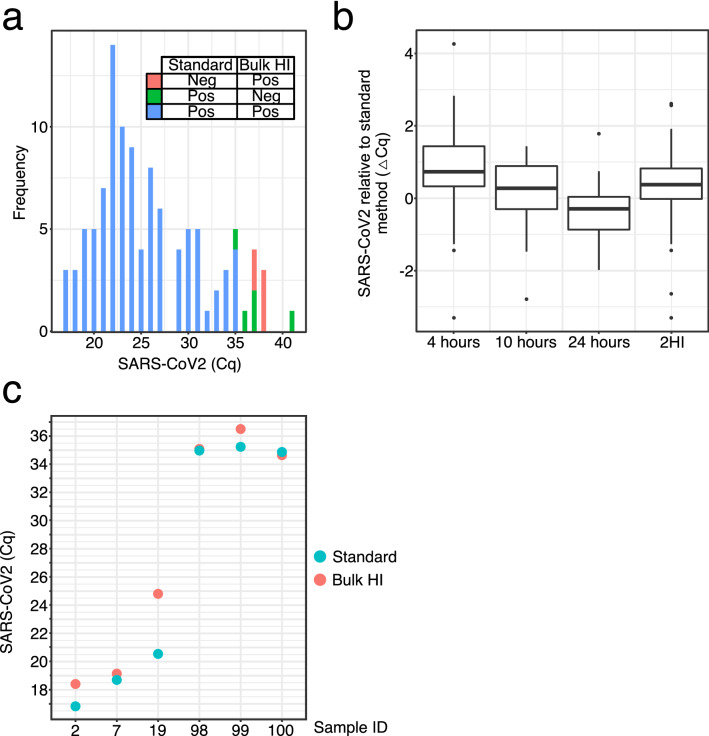
Bulk HI concordance data using clinical OP/NP samples. (**a**) Concordance of SARS-CoV-2 RT-qPCR test results for clinical OP/NP samples which tested positive in either the standard clinical assay or using the bulk HI protocol, and binned according to the clinical assay Cq. (**b**) Distribution of change in Cq value (Clinical Cq—Experimental Cq) versus the clinical test for the primary bulk HI protocol (bulk HI) and variations (deliberate delay of 10 or 24 h post HI, or the fall-back method of a second HI step in the lab (2HI). (**c**) Selected samples from (**a**) showing the impact of bulk HI on RT-qPCR detection is not Cq dependent. Data was plotted using R (v4.1.0 “Camp Pontanezen”) with ggplot2 (v3.3.3) and the figure was assembled using Affinity Designer (1.9.3).

In establishing a bulk HI protocol for viral inactivation, we found that some sample-dependent and unpredictable small loss in assay sensitivity was inevitable. In order to understand if this could be detrimental to the clinical test, we performed a large-scale concordance study in which a representative set of clinical samples were tested in both the standard procedure and the potential new bulk HI workflow in parallel. Three independent experiments were carried out in which clinical OP/NP swab samples were retained following the first step of clinical testing (sample transfer from swab vial), and vials were then re-capped and re-packaged in fresh UN3733 containment corresponding exactly to the original packing (specifically comprising a UN3733 leak proof bag, an inner sealable bag and an absorbent pad). Re-packed samples were subject to the bulk HI protocol (Supplementary Table 1) followed by RNA extraction and RT-qPCR following the CCTC standard procedure with the exception of omitting the laboratory-based HI step following sample lysis. Three independent experiments were run, each using 200–250 randomly chosen clinical OP/ON swab samples. Results were assessed in terms of test result concordance, by comparing the approved diagnostic assay result to the experimental bulk HI protocol test result for each sample (Fig. 3a, Supplementary Tables 4 and 5). The total sample set over the three experiments comprised 630 samples, 105 of which were given a SARS-CoV-2 positive result in the approved diagnostic assay, with RT-qPCR Cq values ranging from 17 to 38. The remaining test results for this sample set were negative (523 samples) or void (i.e. result not valid, 2 samples). Overall 94% of samples achieved the same test result using the bulk HI protocol, which included all but one positive sample with a Cq below 36, and 514 of the Negative samples (Fig. 3a). Positive samples with Cq values of 36 and above were variably detected between the two processes at approximately equal proportions, with 6 samples having concordance results as ‘Positive to Negative’ and 9 samples having results of ‘Negative to Positive’ (Supplementary Table 5). This variation reflects the limit of RT-qPCR assay sensitivity itself, where detection of such very weak positive samples is inherently inconsistent. When weak positive data from Cq 36 and above are excluded from the analysis, an overall concordance of 99% is seen. For samples that were identified as positive in both protocols, the bulk HI protocol Cq level was typically within 2 Cq of the approved assay result (Fig. 3b) and the average change of 0.81+/− 1.08 Cq was very comparable to the value of 0.79 +/− 0.66 Cq seen for samples incubated for 60 min at 65 °C (Supplementary Tables 3 and 4 respectively), as indicated by earlier studies, some samples were seen in which the impact on Cq was greater, with 14 samples out of the 105 positive samples showing a deviation above 2Cq (Fig. 3b). As seen previously the Cq difference is typically independent of the actual Cq value (Fig. 3c and Supplementary Fig. 7), however since reporting of SARS-CoV-2 test results is limited to positive, negative or void, the actual Cq value obtained in the assay has no impact on the reporting of positive samples as is the purpose of the assay. Overall the concordance data using clinical samples demonstrates that the bulk HI protocol is highly comparable to the existing approved assay, detects positive results with the same degree of reproducibility as the existing assay, and is therefore suitable for use as clinical diagnostic test.

In the concordance study, OP/NP swab samples were tested as soon as possible after the HI step of the original clinical test. In reality there was an unavoidable lag time of approximately 1–4 h between the bulk HI step and the OP/NP sample being added to RNA extraction lysis buffer (at which point RNA integrity is preserved). This was due to the logistics of the experiment, i.e. the time it took to repack the sample swab vials and follow the bulk HI protocol. In the real-world environment of a high-throughput COVID-19 Testing Centre, the normal scenario is that samples are processed as quickly as possible and immediately after heat inactivation. However, unforeseen circumstances around general logistics or other incidents may arise and extend this time. Therefore, it was critical to determine the length of time following HI that samples can be processed and still preserve the quality of the diagnostic test. Similarly, as a contingency plan against oven malfunction or operator error that could result in uncertainty on whether bulk HI was successful, a fall-back process was needed. In our case, this would be to revert to the standard assay in which samples are heated in the lab after addition of RNA extraction lysis buffer.

To address the risk of time delay, the concordance study already described above had an additional test group in which the samples being retested were retained and then tested for a second time but after a 10 h delay after the HI cycle. Concordance for this data set was comparable to the samples processed without a delay, i.e. in which the samples were tested within 1–4 h following HI (Fig. 3b, Supplementary Table 5 and Supplementary Fig. S8). In addition, a separate study of 184 clinical OP/ON swab samples was performed in which sample processing occurred 24 h after HI. For these samples we saw the same result in that concordance is preserved for SARS-CoV-2 positive samples below a Cq of 36 (Supplementary Fig. S9, Supplementary Table 5 and Fig. 3b).

To address the risk of a failed HI cycle and the resultant requirement to revert to the standard assay protocol, we carried out further experiments. Here we subjected clinical OP/NP swab samples to the bulk HI protocol, but then followed the standard assay as if no HI cycle had been used. This meant that the samples were heated twice during the workflow (Supplementary Fig. S10). The time taken to do this work meant that samples were retested using the experimental conditions one day after the clinical test. Nonetheless, we saw 88.8% concordance in the test results of the 171 samples processed, and as before, the majority of discordant test results were above a Cq value of 36 (Supplementary Fig. S10).

Finally, at the time this work was carried out, the CCTC was contributing to the genomic surveillance effort to monitor SARS-CoV-2 variants of concern by providing extracted RNA from positive tests to the Wellcome Sanger Institute for sequencing. Sequencing quality was not changed by the bulk HI workflow, as illustrated by an equivalent base coverage prior to and following implementation of the bulk HI protocol at the CCTC (Supplementary Fig. S11).

## Discussion

This is the first report of a high-throughput HI protocol for SARS-CoV-2 testing that is performed on UN733 packaged OP/NP swab samples in bulk. Although the use of heat is an effective means of SARS-CoV-2 inactivation^8,11–13,23–25^, COVID-19 testing laboratories have relied on chemical lysis during RNA extraction for viral inactivation. Chemical lysis is highly effective mechanism of virus inactivation^26,27^, but requires handling of potentially viable SARS-CoV-2 samples that represents a risk to operators. The risk is typically mitigated by the use of a MSC, but this limits testing to facilities having sufficient space and suitable containment. Reliance on the use of MSCs is costly and suffers from poor ergonomics for operators. In our experience, MSC cabinet availability can be a major limiting factor for sample throughput, for example in the event of MSC breakdown or an unplanned increase in the samples.

The ideal solution is to inactivate OP/NP swab samples as the first step in the process. This renders the entire workflow lot safer and the rest of the sample processing can be performed on the open bench without the risk of exposure to viable SARS-CoV-2 virus. We have optimised, validated and implemented such a process using an industrial steam oven originally designed for the catering industry. The bulk HI protocol achieves rapid and consistent heating of packaged samples in bulk, at a rate of 500 samples per hour per oven. A critical aspect of this work has been an experimental demonstration of virus inactivation using the bulk HI protocol on mock OP/NP swab samples. Insufficient exposure at the required temperature could result in incomplete viral inactivation^13,28^ which is especially important for samples with high viral load^11,12,29^. In addition our bulk HI protocol employs wet heat as opposed to dry heat as previously used^30^, which can lead to evaporation and incomplete viral inactivation^28^.

This study is the first to demonstrate successful SARS-CoV-2 detection following a bulk HI of clinical OP/NP swab samples at scale^23,26^. We tested approximately 1200 OP/NP swab samples in concordance studies, in addition to the use of known positive OP/NP swab samples for the supporting studies. The bulk HI protocol resulted in an average increase of less than 1 Cq in the RT-qPCR assay. This was seen as highly comparable to the existing approved assay. We observed several samples in which the Cq value was significantly increased, however this had no effect on the ability to report positive test results below the threshold at which weak positives already suffer from unreliable detection due to the limit of detection of the assay overall. The importance of high Cq positive results is unclear in the context public health^31^ and it has been suggested that prioritising speed and frequency of testing over sensitivity could have a greater impact on infection surveillance^32^. Importantly, the bulk HI protocol had no detrimental impact on the ability to generate SARS-CoV-2 variant sequencing data for genomic surveillance, a critical element of pandemic monitoring and control.

The bulk HI protocol was implemented at the CCTC on the 11th of February 2021 following clinical and DHSC approval. The CCTC employed three ovens running 24 h a day, with a calculated maximum capacity of over 30,000 samples per 24 h period. The working capacity of the CCTC was 22,000 samples per day^7^, well within the bulk HI protocol capacity. Informal feedback from scientists working in the CCTC confirmed the advantages around comfort and peace of mind provided by removing the need for extensive MSC use and handling of inactivated as opposed to potentially viable samples. Although hard to quantify, these benefits are significant, leading to a higher performing workforce employed in a repetitive but high pressure role, particularly when testing demands are high. In the wider context of global testing, a bulk HI protocol may be more feasible where containment level 2 laboratories are less widely available or difficult to establish^26^. Up-front bulk HI is also an ideal solution to the challenges of automated sample handling for SARS-Cov-2 testing. Currently, to enable automated processing, the sample handling equipment needs to be contained in a suitably sized and often bulky safety cabinet, or have equivalent containment as part of the platform itself. This can be a challenge given the large size of automation platforms, the high cost of bespoke containment solutions and the impact on accessibility and ergonomics. Once containment is not required, robotics can be used safely on the open bench in a standard laboratory. This allows the use of any automation platform leading to the realisation of the significant benefits of automation for high throughput testing as a result of reduced errors and higher throughput. The safety and cost benefits of bulk HI, in particular by not having to use MSCs and secondary containment for automation, should help the healthcare sector improve testing and become more efficient, particularly in lower and middle income group countries^33,38^. The use of qPCR based testing is becoming an integral part of international travel. Given the emergence of new virus strains and the widely accepted view on multiple waves of COVID-19, this is not expected to change in near future. The high costs of mandated tests on ordinary travellers can be significant, particularly for families who may have to take 3–6 tests per trip. This places a financial burden on individuals and families already under pressure, particularly in periods after extensive travel restrictions that have prevented normal family and social contact. Our protocol could help reduce costs for these families and may be employed on a much larger scale by service providers for ‘test to travel’ and ‘test to release’.

Finally, achieving SARS-CoV-2 inactivation without the use of chemical means enables workflows that do not involve RNA extraction. For example use of a direct to PCR approach in which heat inactivated samples are added directly to RT-qPCR reactions without prior RNA extraction^7^. The combination of bulk HI and direct to PCR testing is a significant opportunity to simplify the test process, reducing costs and increasing speed and productivity. Cost and speed are crucial aspects to the current COVID-19 response, greatly impacting all from an individual to a global scale. We hope the bulk HI protocol for SARS-CoV-2 testing enables lower risk and consequently highly flexible COVID-19 testing worldwide.

## Materials and methods

### Ethics statement

This study has been performed in accordance with relevant international and local laws and in accordance with The Declaration of Helsinki. This study was conducted as part of the Lighthouse Laboratories surveillance for COVID-19 infections set up under the auspices of section 251 of the National Health Service Act 2006 and/or Regulation 3 of The Health Service (Control of Patient Information) Regulations 2002. The study therefore did not require individual patient consent or ethical approval. No Patient Identifiable information (PII) was received by the Centre. Authors only had access to anonymised data in the form of sample barcodes. Approval for the operation of the CCTC and improvements to the procedures used therein was granted by the Department for Health and Social Care under the emergency provisions granted by the Secretary of State under Section 251 of the National Health Service Act.

### Cell line and virus

Murine 17 clone 1 (17Cl-1) cells were grown in Dulbecco’s Modified Eagle’s Medium (DMEM) low glucose supplemented with 5% (v/v) Foetal Calf Serum, 6% (v/v) TPB, 1 × L-glutamine, 1 × non-essential amino acid and 100 U penicillin ml^-1^ and 100 mg streptomycin ml^-1^ at 37 °C with 5% CO_2_^2, 18^. Recombinant murine hepatitis virus (MHV) strain A59 recovered from a full-length infectious MHV-A59 cDNA clone was received as kind gift from Dr Nerea Irigoyen, University of Cambridge, UK and propagated in 17Cl-1 cells supplemented with 0.2% bovine serum albumin (BSA) and 50 μg/ml DEAE-dextran^18,34,35^.

### MHV-A59 heat treatment

First experiment was performed using cell-free virus stock and heat inactivation in a thermocycler for 5, 10 or 15 min at 60 °C, 65 °C and 75 °C. Subsequently, cell-free virus stock of MHV-A59 (7.9 × 10^7^ TCID_50_/ml, 250 μl) (cell-free) or murine 17Cl-1 cells inoculated with MHV-A59 (infected cells) at MOI of 0.15 TCID_50_ for 24 h (250 μl) were added to VTM-containing swab. Samples were sealed in a UN3373-compatible containment, mimicking biological sample conditions, and were then bulk HI. Two independent experiments assayed by TCID_50_, with 6 or 12 replicates of each condition. In order to mimic the process of handling clinical sample, all samples were prepared elsewhere and transported to the test site at room temperature. At the test site, each set of VTM-samples containing cell-free virus stock or virus-infected cells were heat treated through the Electrolux Oven. Non-heat treated samples remained were carried along with samples used for bulk HI. Following heat treatment, all samples were transported back to the laboratory at room temperature and TCID_50_ infectivity assay were performed immediately.

### Titration of residual infectivity by TCID_50_

Ten-fold serial dilutions of sample following heat inactivation treatments were prepared in DMEM supplemented 2.5% FBS and 3% TPB. Of these dilutions, 50 μl was inoculated onto monolayers of 17Cl-1 cells grown on 96-well plates and incubated at 37 °C a 5% CO_2_ incubator. Virus titers were collected after 48 h p.i. and expressed as TCID_50_ ml^-1^ values by the Reed–Muench method^36^.

### Detection of SARS-CoV-2 in OP/NP swab samples by RT-qPCR

The CCTC standard SARS-CoV-2 diagnostic RT-qPCR assay is described in detail elsewhere^7^. Briefly, clinical OP/NP samples from various sources (Home Test kits, Care Homes, Regional Testing Centres, Border tests) were received at the CCTC in leakproof UN3373 packaging containing a screw capped sample tube with a swab stick immersed in viral transport medium (VTM). OP/NP sab vials were unpacked and racked within a MSC and following Containment Level 2 precautions. For each sample, 200 µl of VTM was transferred to a 96 deep well sample plate, followed by the addition of RNA extraction lysis buffer, Proteinase K and Internal Extraction Control (IEC) RNA. The sample plate was sealed and double contained, and placed at 65 °C for 10 min followed by 10 min at room temperature. From this point onwards the process was performed outside of MSC in Containment Level 1 laboratories, since potentially viable viral material has been inactivated by guanidine isothiocyanate (GITC)-containing lysis buffer and heat. RNA was extracted using the RNAdvance Viral Kit (C63510) on Biomek i5 or i7 automated platforms from Beckman Coulter. Viral RNA was eluted in nuclease-free water and then used for RT-qPCR using the Genesig® Real-Time COVID-19 PCR High Throughput assay kit (Primer Design Ltd, Geneisg Z-Path-COVID-19-CE HT1.0) as described by the manufacturer except that 10 µl (i.e. half) reaction volumes were used. RT-PCR reactions were prepared in white, 384-well LightCycler 480 Multiwell Plates (Roche #04729749001), 6 µl RT-qPCR master mix was added using a ThermoFisher Multidrop Combi and 4 μl extracted RNA sample was added using an Agilent Bravo. RT-qPCR reactions were run on a Light Cycler 480 II and data was analysed using a bespoke algorithm (FastFinder software, UgenTec) to define Cq values and assign test results following interpretation of controls (positive, negative, IEC) and according to a defined decision tree.

For supporting studies exploring HI and the bulk HI concordance experiments, OP/NP samples were tested as above and retained after the point of transferring VTM to 96 well plate transfer. This typically left 0.8–2.8 ml VTM behind, depending on the swab kit design. A thermal cycler was used for the experiments exploring temperatures above 65 °C, and sample volume was reduced to 100 µl to allow this. For bulk HI of OP/NP samples, the swab vials were repacked into double containment to replicate unopened clinical tests. Sample bags were loaded into the oven trays at 25 per tray and 10 trays per oven. All experiments with the industrial oven contained 250 samples, made up with mock (i.e. clean assembled OP/NP swab test kits) as needed. Calibrated thermocouple vials were included in all runs at 2 per tray to monitor heating, and in all cases the temperature readings were as expected according to the profiles seen in the oven validation experiments. Following bulk HI, samples were allowed to cool to a safe handling temperature and had reached room temperature by the time of pipetting.

### Statistical analysis and software

The key response measure in these analyses is Cq, and in all cases the Cq data were found to be approximately Normally distributed on visual inspection of the distribution, which fits with scientific interpretation. Cq data should be averaged based on the arithmetic mean, and differences in Cq can be used to calculate the gene expression ratio^37^. So in all summary tables, the arithmetic mean and standard deviation of Cq were presented. JMP 16 was used to perform statistical analyses of the data. Main data was plotted using R (v4.1.0 “Camp Pontanezen”) with ggplot2 (v3.3.3) and the figures assembled using Affinity Designer (1.9.3). Supplementary data was presented using GraphPad Prism 8 and Tibco Spotfire (version 7). Supplementary Tables 2, 3 and 4 provide summary statistics and 95% confidence intervals for the comparisons made. While these confidence intervals show a small statistical bias, in magnitude the differences are very small.

## Supplementary Information

Supplementary Information.
