# Supplementary material for: Heat inactivation of clinical COVID-19 samples on an industrial scale for low risk and efficient high-throughput qRT-PCR diagnostic testing
Source: Sci Rep. 2022 Feb 21;12:2883. doi: 10.1038/s41598-022-06888-z (PMC8861189; doi:10.1038/s41598-022-06888-z)
Supplement: Supplementary file 1 — Supplementary Information. [file 41598_2022_6888_MOESM1_ESM.docx]

**Delpuech et al., Bulk Heat Inactivation Supplementary Figures.**

**Supplementary Figure S1:** Sample racking and trolley system used for bulk HI using in the Electrolux Skyline Combi Oven. Lower left image shows samples in UN3373 packaging placed in a tray (upper left) ready for loading onto the trolley (right).


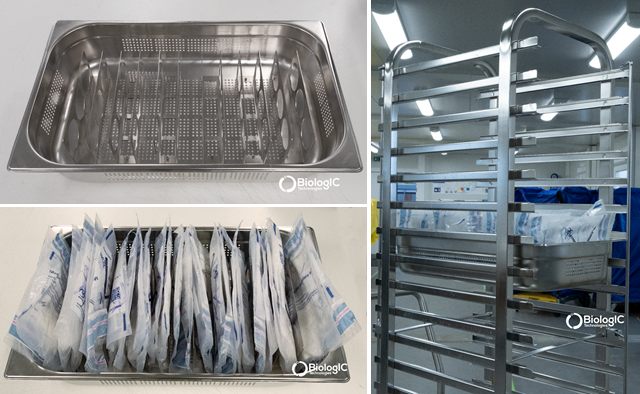


**Supplementary Figure S2:** Bulk HI cycle optimisation using the Industrial Steam oven, Experiments 2 and 3. Experiment 1 is shown in **Fig. 1c**. Twenty thermocouple containing swab vials were placed throughout a full oven load of mock samples and temperate monitored during execution of a HI cycle. All thermocouple vials achieve the intended viral inactivation conditions of 65°C for 10 min and no vial is heated beyond approximately 77°C.

Experiment 2:


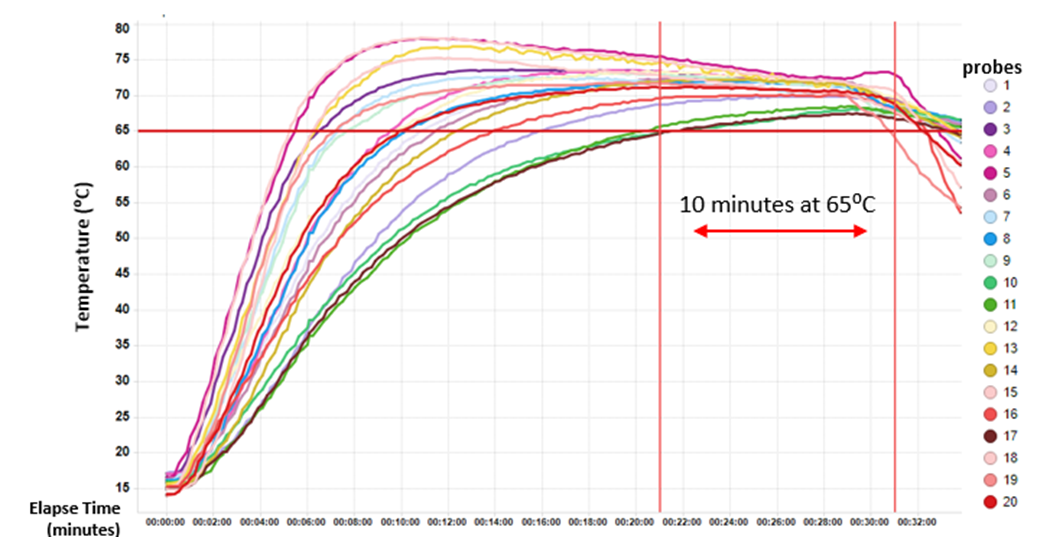


Experiment 3:


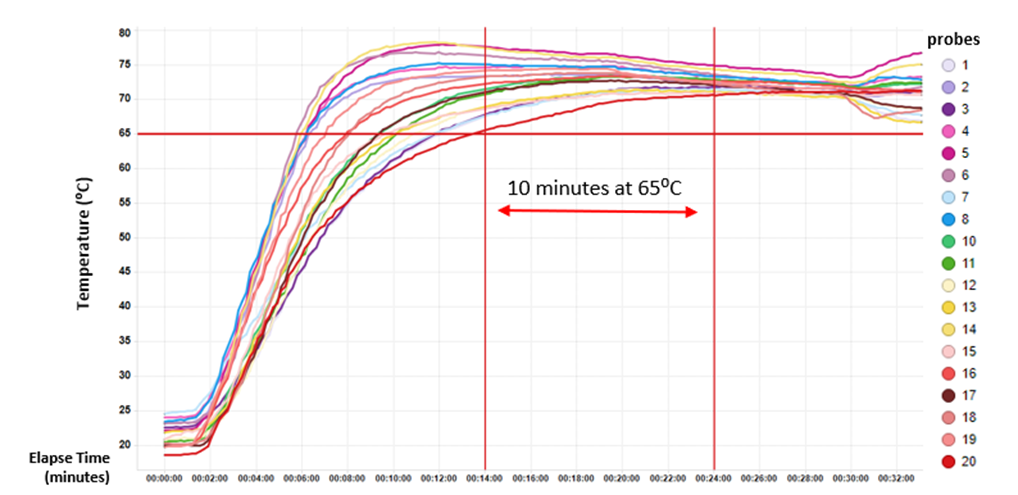


**Supplementary Figure S3:** Validation of the HI cycle using different vial types.

Typical OP/NP swab vials in use and the UN3373 outer bag used for containment:


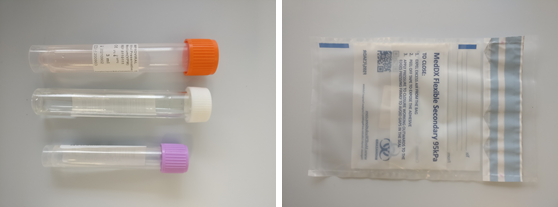


Temperature data from thermocouple containing swab vials placed throughout a full oven of mock samples during execution of a HI cycle. The Red Vacutainer type showed the potential for a significantly different heating profile to other vials in the same run, although this was within the heating range of vial on the whole seen in other experiments. Notably however, several of this vial type were seen to warp after the HI cycle so were excluded from the bulk HI process using this HI cycle.


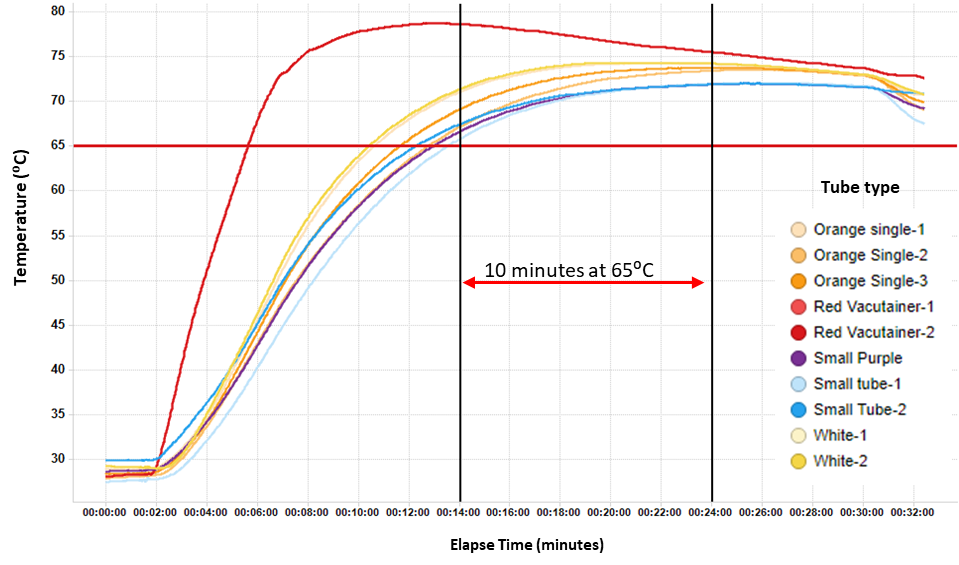


**Supplementary Figure S4:** Validation of the HI cycle using different OP/NP swab vials containing multiple swabs. Thermocouple-containing vials with single or 5 swabs (multi) were placed throughout a full oven of mock samples and temperate monitored during execution of a HI cycle. The number of swabs does not alter the heating of samples outside the typical range of that seen in single swab vials.


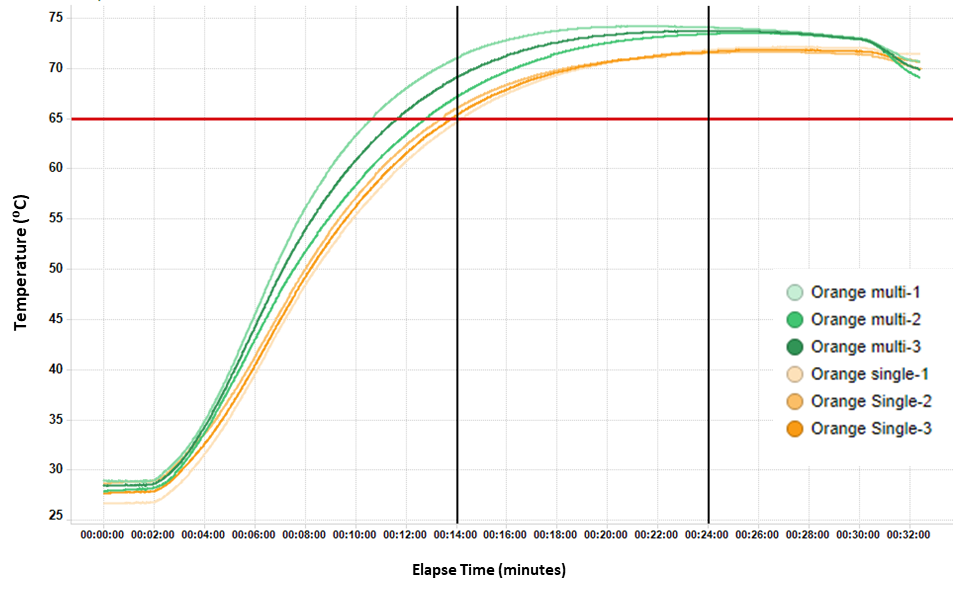


**Supplementary Figure S5:** Variability of SARS-CoV-2 detection. Clinical OP/NP swab samples (23) were tested over 3 independent days (E1,E2,E3). Dots represent replicate (n=2 or n=3) measurements of each sample on the same day. ‘T65’ is 65°C in a thermocycler, ‘65C’ is 65°C the standard oven. The SD of the raw data within each sample/temperature appeared to show no linear dependency of SD on mean. Therefore, a single estimate of the measurement variability was derived, representative across the range of mean values found. An Analysis of Variance model was fitted to include sample ID and temperature as factors, and the residual variability was used to estimate the pooled within-sample variability (0.33Cq).


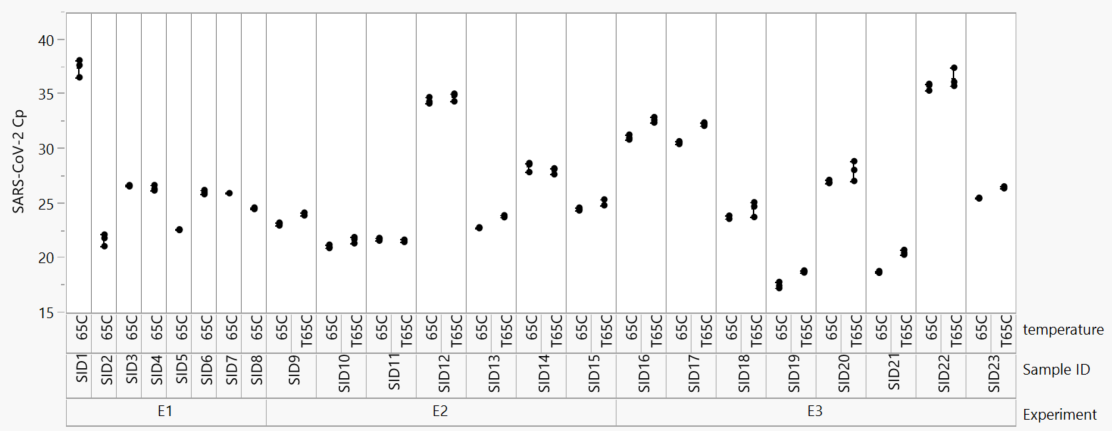


**Supplementary Figure S6:** SARS-CoV-2 detection by RT-qPCR following incubation at 65°C for 20 min, 60 min or 90 min (y-axis) compared to 10 min (x-axis). Each point represents a single sample. The diagonal line is the line of agreement. Points falling on this line indicate precise agreement between the two incubation times. The 20 min plot indicates very close agreement, while the 60 min and 90 min plots show a small bias with the lengthened incubation times leading to slightly higher Cq values. These data were analysed by paired t-test to calculate a 95% confidence interval for the difference between each lengthened incubation time (20, 60, 90 min) vs the control incubation time of 10 min (Supplementary Table 3).


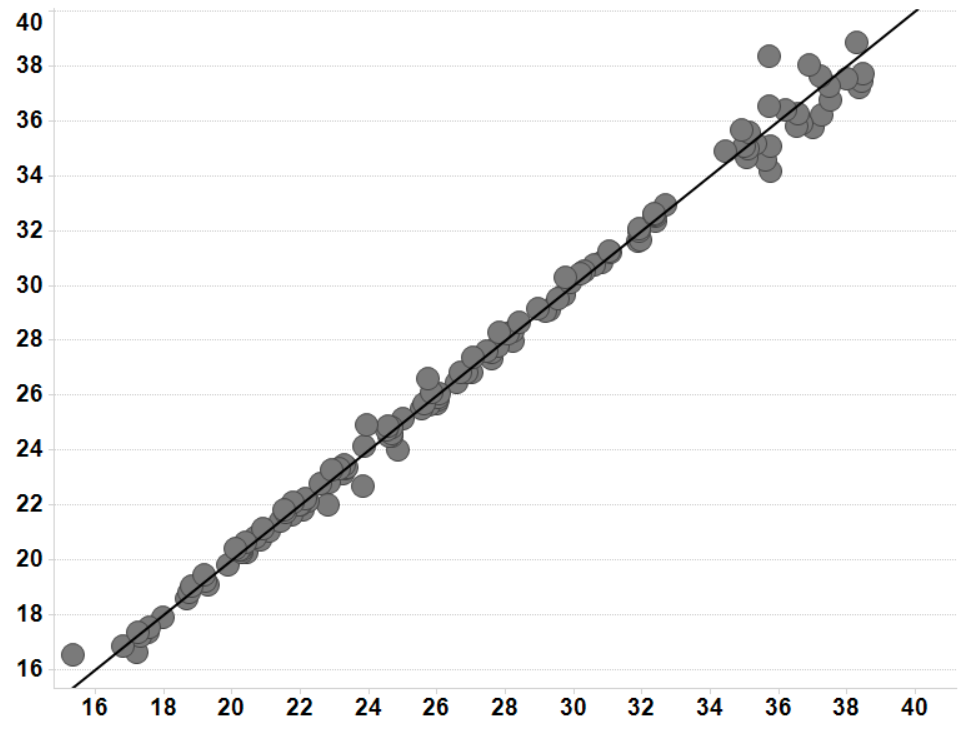


**SARS-CoV-2 Cq (10 minutes)**

**SARS-CoV-2 Cq (20 minutes)**


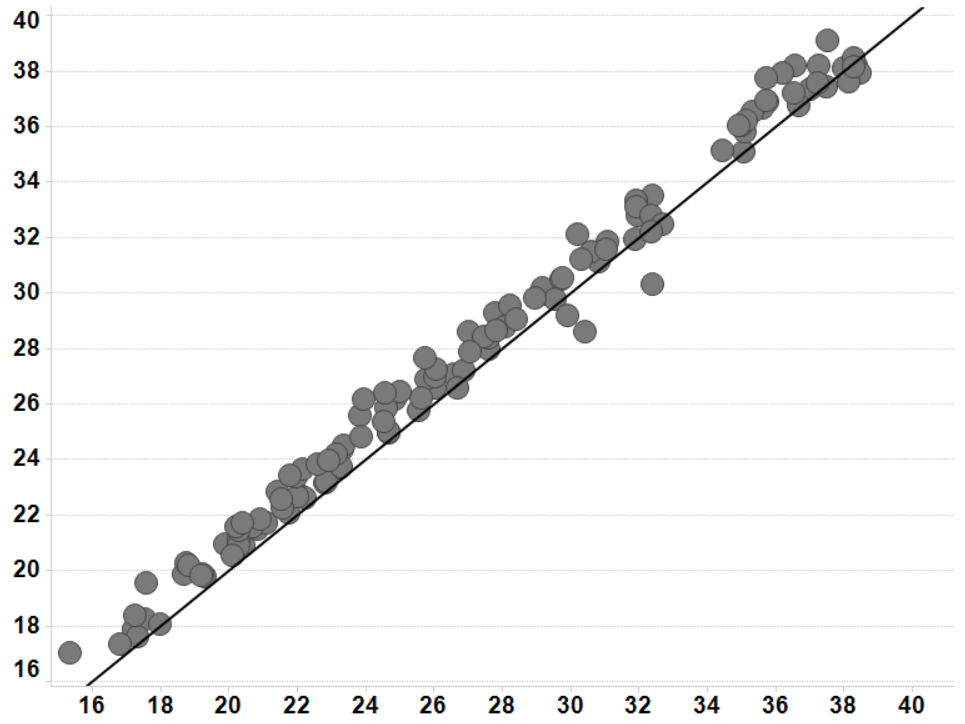


**SARS-CoV-2 Cq (10 minutes)**

**SARS-CoV-2 Cq (60 minutes)**

**minutes**


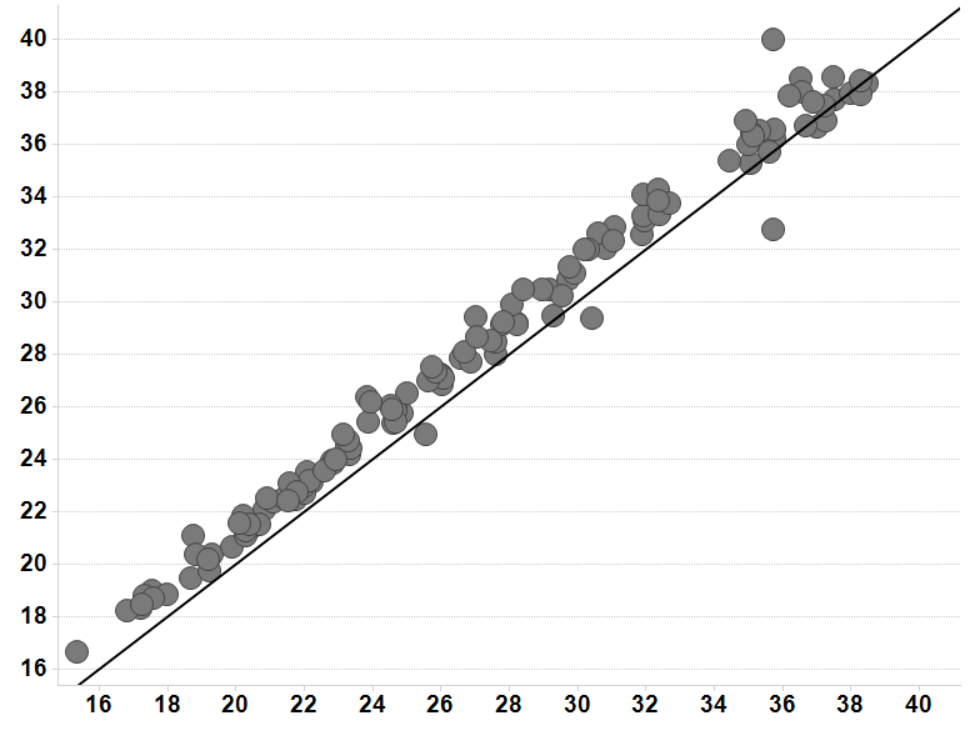


**SARS-CoV-2 Cq (10 minutes)**

**SARS-CoV-2 Cq (90 minutes)**

**Supplementary Figure S7**: Cq values from concordance studies of clinical OP/NP swab samples (n=100) using the bulk HI protocol compared to the standard approved process. Each point represents a single sample. The diagonal line is the line of agreement between the ‘Bulk HI’ Cq value and the ‘Standard process’ Cq value.


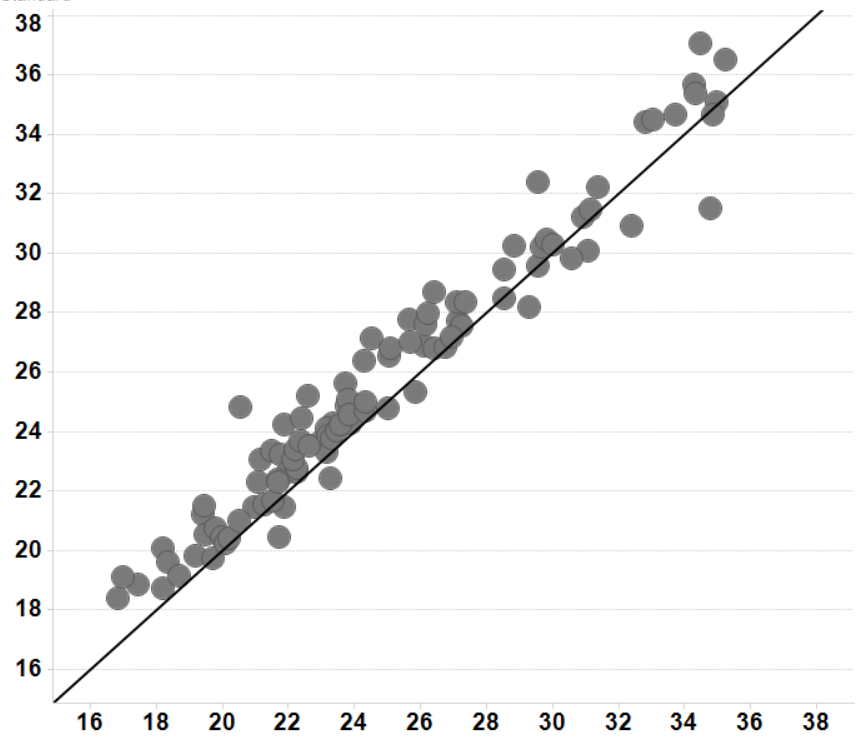


**SARS-CoV-2 Cq (Standard process)**

**SARS-CoV-2 Cq (Bulk HI)**

RT-qPCR data for selected samples using each protocol. Green circles show the standard approved process, orange starts show the bulk HI protocol with sample processing withing 1 to 4 hours, pink triangles show the bulk HI protocol with sample processing after 10 hours.


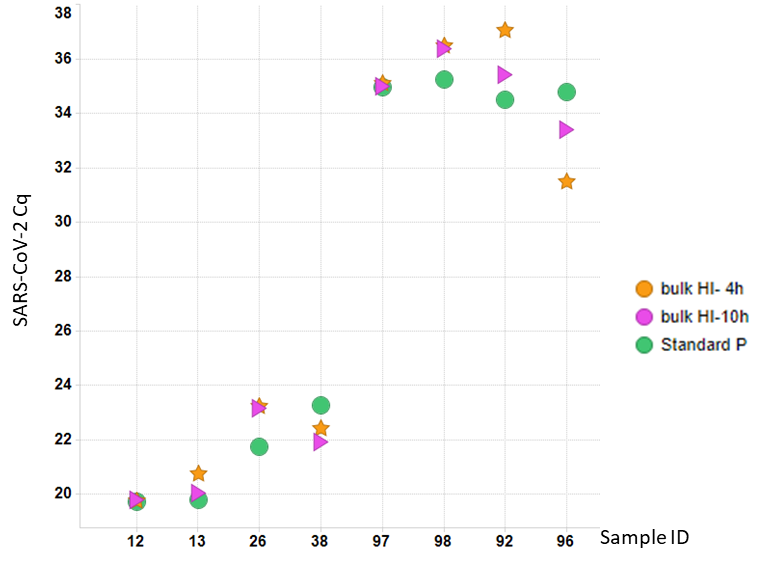


**Supplementary Figure 8:** Concordance data of clinical OP/NP swab samples using the bulk HI protocol compared to the standard approved process.

Test result concordance with a delay of up to 4h or 10h following bulk HI. Samples are categorised as ‘Pos to Pos’ (Green; Positive in clinical test and positive in bulk HI test) or ‘Pos to Neg’ (Red; Positive in clinical test and negative in bulk HI test), and binned according to Cq value.


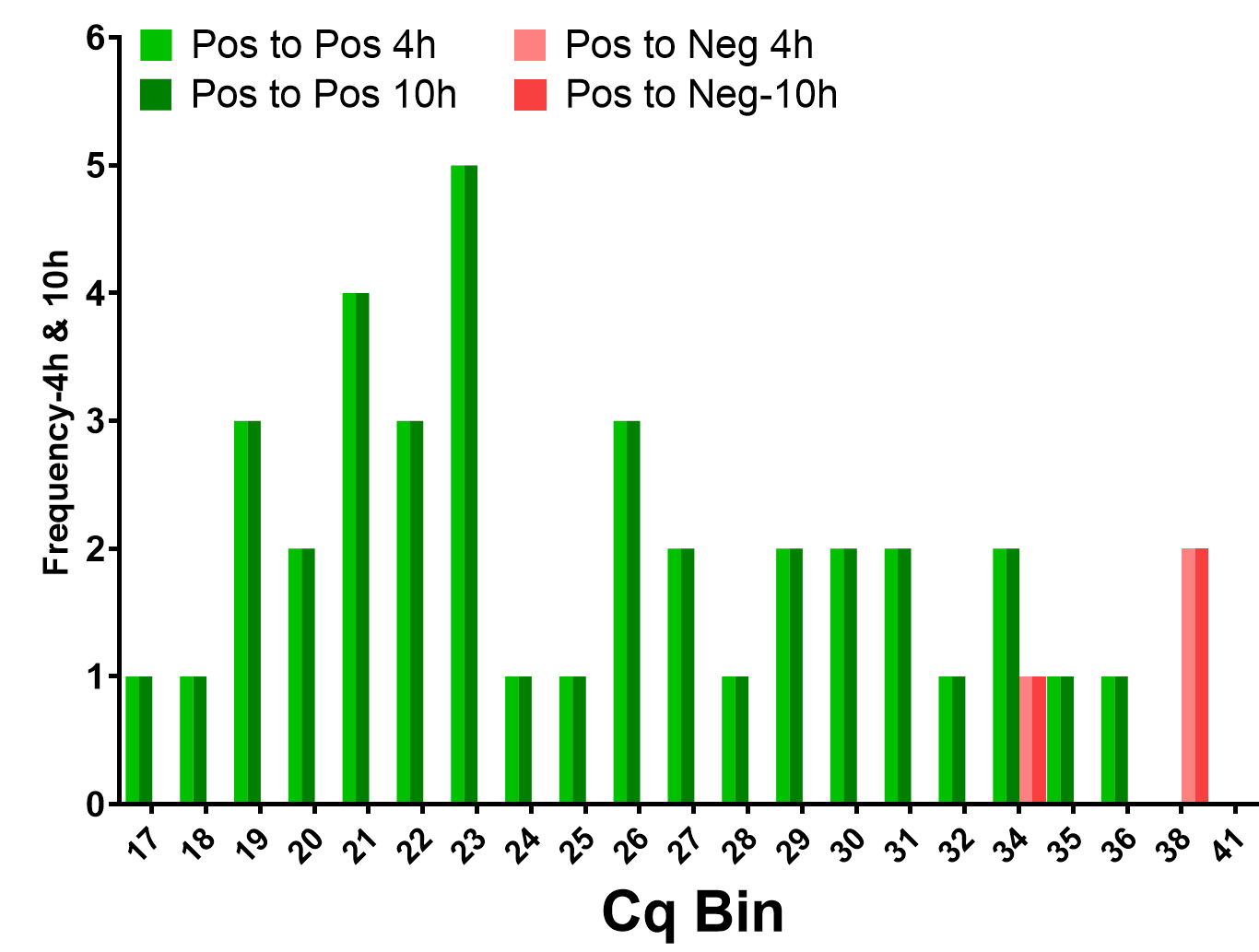


Correlation in Cq values with a delay of up to 4h or 10h following bulk HI.


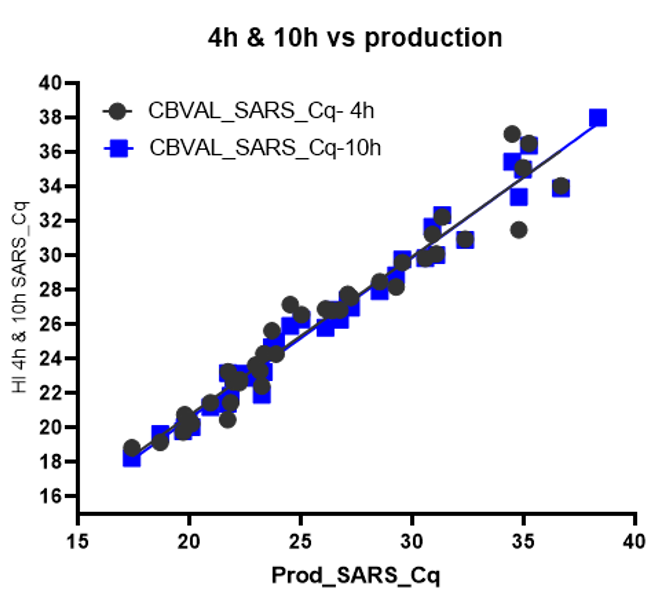


**Supplementary Figure S9:** Test result concordance with a delay of up 24h following bulk HI. Samples are categorised as ‘Pos to Pos’ (Green; Positive in clinical test and positive in bulk HI test), ‘Pos to Neg’ (Red; Positive in clinical test and negative in bulk HI test), or ‘Neg to Pos’ (Blue; Negative in clinical test and positive in bulk HI test) and binned according to Cq value.


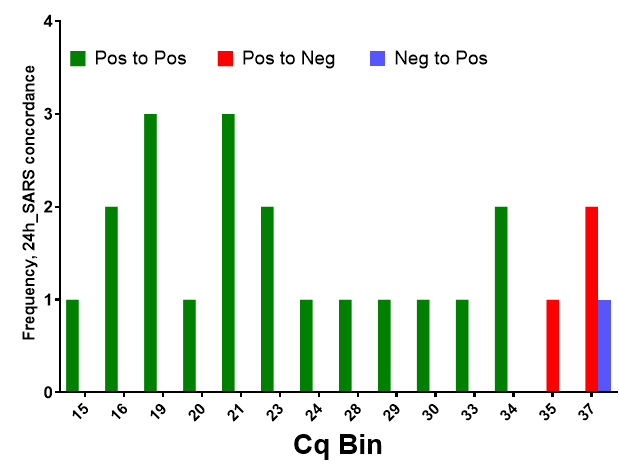


**Supplementary Figure S10**: Test result concordance following the above fall-back process of employing a second heat step after sample lysis.


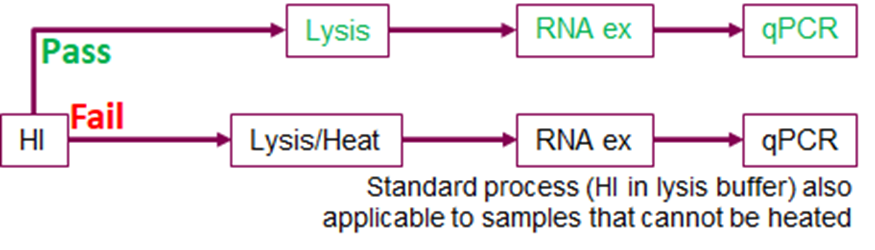


Samples are categorised as ‘Pos to Pos’ (Green; Positive in clinical test and positive in bulk HI test), ‘Pos to Neg’ (Red; Positive in clinical test and negative in bulk HI test), or ‘Neg to Pos’ (Blue; Negative in clinical test and positive in bulk HI test) and binned according to Cq value.


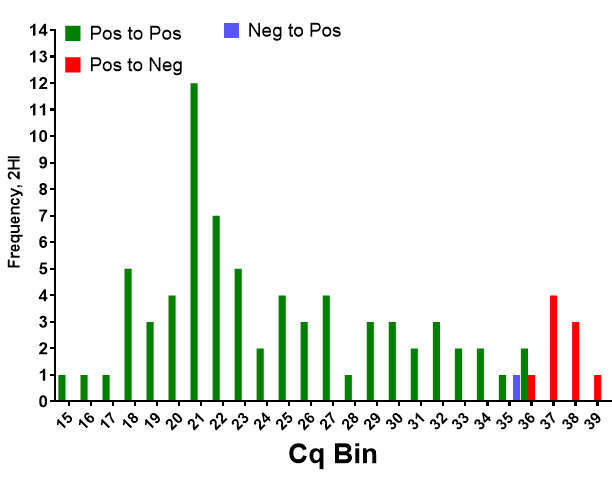


**Supplementary Figure S11:** Sequencing coverage was not affected by the implementation of bulk HI at the CCTC (2_after) compared to before implementation (1_before).


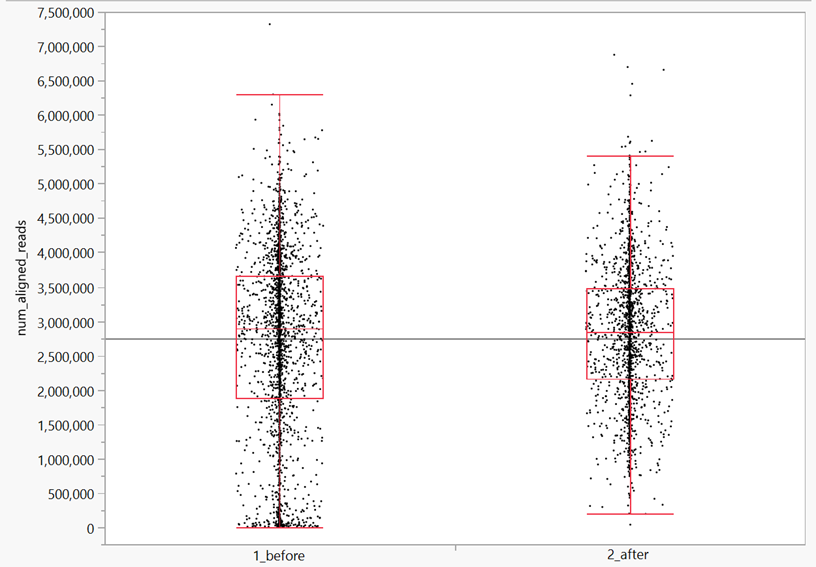


**Supplementary Table1:** The optimised oven program for bulk HI. The oven is preheated prior to sample loading, then ramping temperatures are used to rapidly heat the samples while minimising overheating. A dry step is included at the end to reduce moisture on the out bags and make them easier to handle in the lab.

| **Step** | **Temp °C** | **Humidity (%)** | **Time (min)** |
| --- | --- | --- | --- |
| *Preheat* | 85 | n/a |  |
| *1* | 85 | 99 | 5 |
| *2* | 75 | 99 | 13 |
| *3* | 70 | 99 | 10 |
| *4* | 70 | 0 | 2 |

**Supplementary Table 2:** Effect of temperature on SARS-Cov-2 detection by RT-qPCR. The mean and standard deviation (SD) of the difference in Cq value obtained using the higher temperature versus 65°C is shown.

|  | **Change in Cq at 75°C vs 65°C**  Cq (75°C – 65°C) | **Change in Cq at 80°C vs 65°C**  Cq (80°C – 65°C) |
| --- | --- | --- |
| **Mean** | 0.39 | 0.69 |
| **SD** | 0.4 | 0.55 |
| **N** | 43 | 49 |
| **Lower 95% Confidence Interval for mean** | 0.27 | 0.53 |
| **Upper 95% Confidence Interval for mean** | 0.52 | 0.85 |

**Supplementary Table 3:** Effect of incubation time at 65°C on SARS-Cov-2 detection by RT-qPCR. The mean and standard deviation (SD) of the difference in Cq value obtained using the longer time versus 10 min is shown.

|  | **Change in Cq at 20 min**  Cq (20min-10min) | **Change in Cq at 60 min**  Cq (60min-10min) | **Change in Cq at 90 min**  Cq (90min-10min) |
| --- | --- | --- | --- |
| **Mean** | 0.01 | 0.79 | 1.10 |
| **SD** | 0.50 | 0.66 | 0.75 |
| **N** | 128 | 127 | 129 |
| **Lower 95% Confidence Interval for mean** | -0.07 | 0.67 | 0.96 |
| **Upper 95% Confidence Interval for mean** | 0.10 | 0.91 | 1.23 |

**Supplementary Table 4:** Effect of bulk HI protocols on SARS-Cov-2 detection by RT-qPCR concordance studies. The mean and standard deviation (SD) of the difference in Cq value obtained using the bulk HI protocol versus the standard approved process is shown.

|  | **4h**  Cq (4h-Std) | **10h**  Cq (10h-Std) | **24h**  Cq (24h-Std) | **Fall back HI process**  Cq (Fall back HI-Std) |
| --- | --- | --- | --- | --- |
| **Mean** | 0.81 | 0.19 | -0.60 | 0.19 |
| **SD** | 1.08 | 0.84 | 0.85 | 0.85 |
| **N** | 100 | 54 | 19 | 54 |
| **Lower 95% Confidence Interval for mean** | 0.59 | -0.04 | -1.01 | -0.04 |
| **Upper 95% Confidence Interval for mean** | 1.02 | 0.42 | -0.19 | 0.42 |

**Supplementary Table 5:** Bulk HI concordance data summary.
